# Enhanced Tribological Properties of Vulcanized Natural Rubber Composites by Applications of Carbon Nanotube: A Molecular Dynamics Study

**DOI:** 10.3390/nano11092464

**Published:** 2021-09-21

**Authors:** Fei Teng, Jian Wu, Benlong Su, Youshan Wang

**Affiliations:** 1 Center for Rubber Composite Materials and Structures, Harbin Institute of Technology, Weihai 264209, China; 20S130266@stu.hit.edu.cn (F.T.); subenlong@hit.edu.cn (B.S.); wangsy@hit.edu.cn (Y.W.); 2 National Key Laboratory of Science and Technology on Advanced Composites in Special Environments, Harbin Institute of Technology, Harbin 150001, China

**Keywords:** aircraft tire, CNT/VNR composites, friction, MD simulation

## Abstract

Tribological properties of tread rubber is a key problem for the safety and durability of large aircraft tires. So, new molecular models of carbon nanotube (CNT) reinforced vulcanized natural rubber (VNR) composites have been developed to study the enhanced tribological properties and reveal the reinforced mechanism. Firstly, the dynamic process of the CNT agglomeration is discussed from the perspectives of fractional free volume (FFV) and binding energy. Then, a combined explanation of mechanical and interfacial properties is given to reveal the CNT-reinforced mechanism of the coefficient of friction (COF). Results indicate that the bulk, shear and Young’s modulus increase with the increasement of CNT, which are increasement of 19.13%, 21.11% and 26.89% in 15 wt.% CNT/VNR composite compared to VNR; the predicted results are consistent with the existing experimental conclusions, which can be used to reveal the CNT-reinforced mechanism of the rubber materials at atomic scale. It can also guide the design of rubber material prescription for aircraft tire. The molecular dynamics study provides a theoretical basis for the design and preparation of high wear resistance of tread rubber materials.

## 1. Introduction

Natural rubber is widely used in industrial products due to its excellent elasticity and mechanical properties, such as tires and seals. However, higher requirements are being placed on the natural rubber due to the harsh working condition of aircraft tire. Carbon-based fillers such as graphene (GE) and carbon nanotube (CNT) have been widely applicated in rubber nanocomposites due to the unique structural characteristics, excellent thermodynamic and electromagnetic properties. It has been proved that carbon-based nanofillers can effectively improve the performance of composites, which are suitable for more industrial environments, such as electrical shielding and heating equipment, medical equipment and aircraft tires [1,2].

The properties and potential applications of nanocomposites can be greatly enhanced and expanded by carbon-based nanofillers [3,4,5,6]. Well dispersed epoxidized natural rubber/carbon black (ENR/CB) composite with CNT contained was prepared for high-performance flexible sensors [7]. Bokobza et al. [8,9,10,11,12,13] comprehensively studied the reinforcing effect of CNT on styrene-butadiene rubber (SBR) in mechanical, thermal and electrical properties. The enhancement effect of aminosilane-functionalized carbon nanotube on NR and ENR has been discussed by Shanmugharaj et al. [14,15]. CNT was also used as a model filler for SBR to prepare tightly bound rubber material [16]. The excellent polymer-filler interaction of functionalized CNT was confirmed that it can greatly improve the overall performance including mechanical, thermal and electrical properties of the NR/CNT and ENR/CNT nanocomposites [17,18]. These fillers are regarded as ideal materials in aircraft tire applications to improve the strength [19], modulus [20] and wear resistance [21] of natural rubber materials. Atieh et al. [22] found that the Young’s modulus of the SBR/CNT composite containing 10 wt.% CNT was six times higher than that of the pure SBR due to the excellent strength and interfacial effects of CNT. Compared with other fillers, carbon-based fillers can achieve better reinforcement effects with a smaller dosage, thereby reducing pollution and further improving material performance [23]. Kumar and Lee [24] studied the influence of CB and CNT on the Young’s modulus of filled silicone rubbers (SRs). Results showed that the Young’s modulus of CNT-filled SR also increased from 272% to 706% when CNT was added from 2 phr to 8 phr, which is much larger than 125% with 10phr CB-filled SR. In addition, the different content of carbon-based fillers also has different reinforcement effects on the rubber matrix. Many research works [7,25,26,27,28,29,30] have proved that excessive CNT can cause agglomeration of fillers, which leads uneven dispersion and affecting the properties of composite materials.

It is confirmed that cross-linking vulcanization is one of the most important reasons for the excellent elasticity and deformation recovery of rubber materials [31,32,33,34,35,36,37,38]. The degree of crosslinking and cross-linking bond type greatly affect the overall properties of vulcanized rubber. The accelerator is often used to increase the degree of crosslinking of vulcanized rubber, thereby reducing pollution and improving material properties [39]. Sainumsai et al. [40] and Fan et al. [41] both tested vulcanized natural rubbers (VNRs) made by conventional vulcanization (CV), semi-effective vulcanization (SEV) and effective vulcanization (EV) methods. The crosslinking density and the content of monosulfidic, disulfidic and polysulfidic crosslinks were obtained. It was indicated that the distributions of sulfur crosslink types effect the strain-induced crystallization and dynamic mechanical properties of vulcanized rubber.

The enhancing mechanism of rubber composites by CNT, GE and other common reinforcing fillers were explained via molecular dynamics (MD) simulations [42,43,44,45]. In particular, it can reveal the micro-reinforcement mechanism of carbon-based fillers and their functionalized products on the polymer matrix [46,47,48,49,50,51,52]. The interface interaction between CNT and polymer matrix was studied by molecular simulation [53,54,55]. It was found that the pull-out force of CNT and the elastic modulus of the matrix are both affected by the diameter of nanotube, however, the shear strength of the interface is mainly affected by the length. In addition, the cross-linked structure of polymers can be developed by MD models, such as epoxy resin and vulcanized rubber [56,57]. Zhang et al. [58] developed a vulcanized SBR molecular model to compare the tribological properties of vulcanized SBR and SBR. It was verified that vulcanization can improve the tribological and interfacial properties of rubber materials at the atomic scale.

In summary, the performance of rubber materials is influenced by the vulcanized cross-linked structure and carbon-based filler. The reinforcement is also affected by crosslinking degree, distribution of crosslinking bond types and dosage of carbon-based fillers. The enhancement mechanism has been studied from the perspective of vulcanization and nanofillers at the atomic level [59,60]; however, it mainly focusses on the oligomers and resin materials rather than rubber materials. In addition, the crosslinking degree, distribution of vulcanization bond types and CNT dosage have not been specific described in the existing studies at atomic scale.

A series of VNR atomic models reinforced by different content CNT were developed to reveal the mechanism of CNT-reinforced VNR. A new VNR model was developed with considering the distribution of sulfur bonds and crosslinking degree. As the main component of road surface, SiO_2_ can be regarded as the direct contact material with tires. As results, the interfacial interaction between SiO_2_ and VNR plays a significant guiding role in aircraft tire rubber materials design and evaluation. Here, the reinforced CNT/NR models and the CNT/NR-SiO_2_ interface models were developed to study the CNT reinforce mechanism on the vulcanized NR and the atomic behaviors of the composites on the friction interface.

## 2. Molecular Dynamics Model

MD models were developed by Materials Studio Software (MS). Firstly, the NR molecular chain, sulfides for the synthesis of crosslinking bonds, CNT and 45 Å^3^ empty periodic cell box were obtained. Then, different numbers of CNT were placed at the center of multiple cell boxes to represent different CNT dosage. The Amorphous Cell Calculation of MS was used to pack 10 NR molecular chains built with 70 repeat units into the periodic cell box by Monte Carlo method. In addition, a variety of sulfides used to generate vulcanized crosslinks were also added to the periodic box in certain proportions and quantities according to the existing experimental measurement results to obtain uncross-linked CNT/VNR models. Next, all the sulfides were used to generate cross-linked bonds by the cross-linking script, then, the cross-linked CNT/VNR composite models were developed. Finally, the silica (quartz glass) model in MS database was imported to build supercell as friction layer. The Cleave surface and the Super cell commands were used for 45 Å × 45 Å × 10.8 Å SiO_2_ supercell and interfacial interaction models were constructed by the cross-linked CNT/NR composite and obtained SiO_2_ slab for further calculation and analysis.

**Figure 1:**
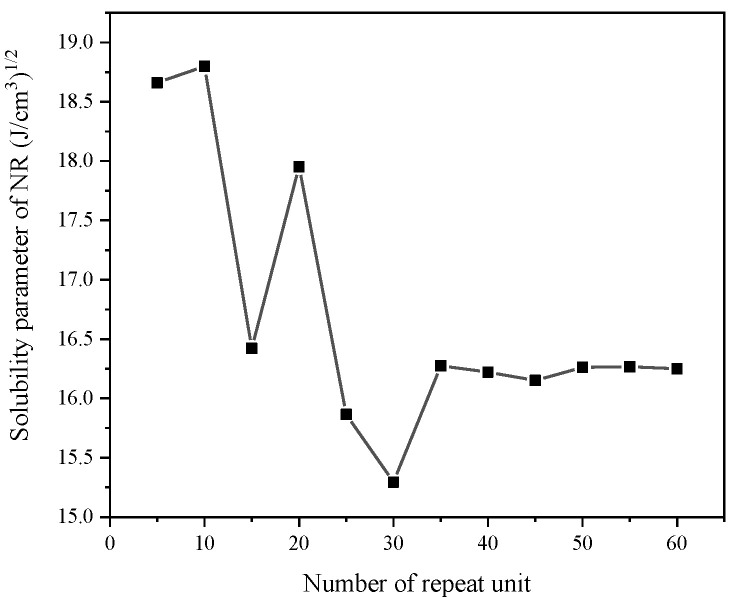
Solubility parameters NR with different degree of polymerization.

The degree of polymerization represents the number of repeating units in a single molecular chain in MD simulation. Longer chain can improve the simulation accuracy but reduce the speed. Therefore, the solubility parameters of single molecular chains with different numbers of repeating units were calculated for proper NR chain length. As shown in Figure 1, the solubility parameter of NR chain begins to stabilize while the number of repeat unit larger than 35, which means the number of repeat units should larger than 35. Moreover, the solubility parameter of NR stabilizes at 16.25 (J/cm^3^)^1/2^, which is consistent with the experimentally measured value of 16.2~17 (J/cm^3^)^1/2^. Here, we built NR chain containing 70 repeat units.

**Figure 2:**
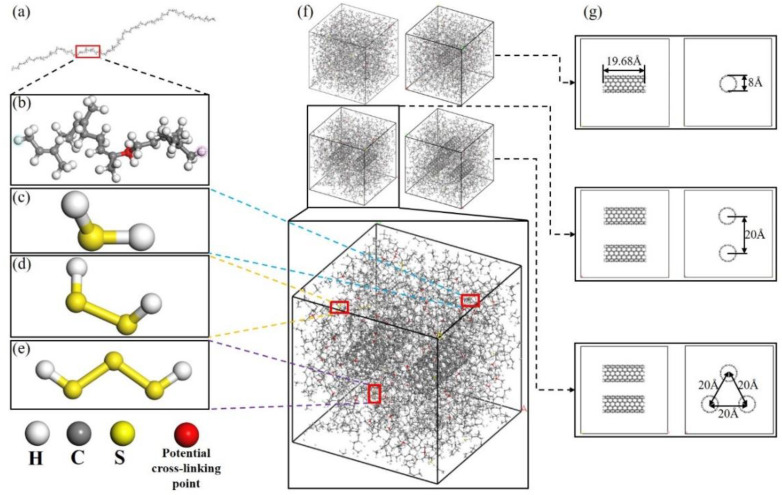
Establishment of un-crosslinked CNT/VNR molecular models. (**a**) NR molecular chain. (**b**) Five repeating units with one potential cross-linking point. (**c**) Monosulfide. (**d**) Disulfide. (**e**) Polysulfide. (**f**) Different CNT contents un-crosslinked CNT/VNR molecular models. (**g**) Size and corresponding positions of the nanotubes.

The modelling of uncross-linked CNT/VNR is shown in Figure 2. Hydrogen atoms were added at both ends for saturated CNT to eliminate the end-side effects. The size and the corresponding positions of the CNT in different CNT content periodic cells are shown in Figure 2g. The crosslink density is defined by:

(1) $$\rho =v/N_{0}$$

where v is the number of crosslinked repeat units and $N_{0}$ is the total number of repeat units.

There is one crosslinked repeat unit in every 50~100 units for conventionally vulcanized rubber materials, which the ρ is about 1~2%. Here, considering the possibility of self-crossing, the number of cross-linking points is determined to be 10 in the model. The ρ of the obtained model is about 2.5%. Experiment results also indicate that the ratio of monosulfidic, disulfidic and polysulfidic crosslinks in the vulcanized natural rubber was about 5:3:2 [40]. Accelerators that promote the cleavage of polysulfide bonds were blended with rubber materials in actual production by EV method, which makes it difficult to form long polysulfide bonds in vulcanized natural rubber. Therefore, vulcanization bonds containing three sulfur atoms were used in this model to characterize polysulfidic crosslinks. Different types of sulfides with the ratio of 5:3:2 and 10 NR molecular chains were introduced to the periodic boxes by the Amorphous Cell Calculation module, which is used to obtain uncross-linked CNT/VNR models with a predefined density of 0.93 g/cm^3^.

**Figure 3:**
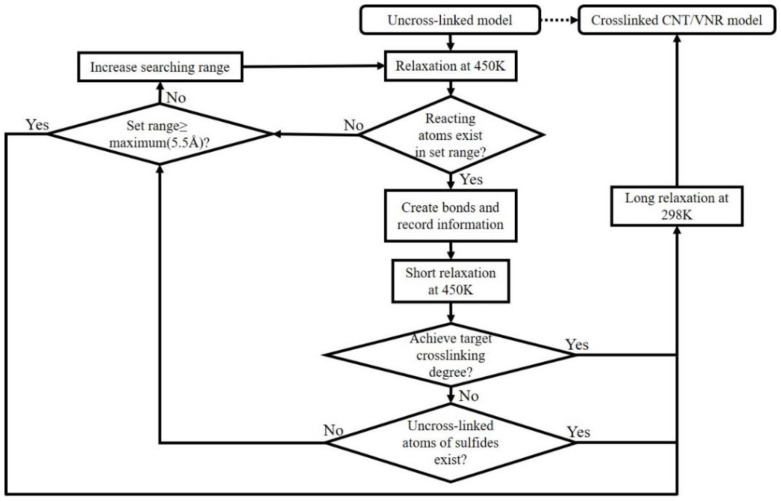
Flow chart of the programing of vulcanization process.

**Figure 4:**
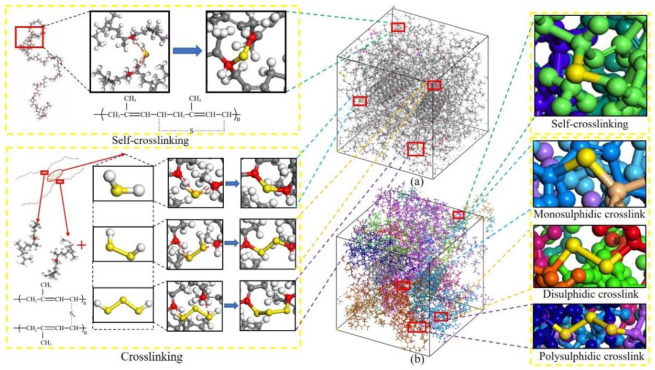
10 wt.% CNT/VNR molecular model crosslinking process and containing crosslinking bonds. (**a**) Colored by atom types as same as Figure 2. (**b**) Colored by molecular chains.

Uncross-linked CNT/VNR models were further used to generate crosslinked structure by crosslinking Perl script at temperature of 450 K. The flow chart of the programing of vulcanization process was shown in Figure 3. As shown in Figure 2b, potential cross-linking points were set in every five repeating units to avoid two cross-linking points being too close. The carbon atoms on the NR chains and sulfur atoms on the sulfides were used to form carbon–sulfur (C-S) bonds when there was sulfide molecular in the range of 1.5~5.5 Å between two potential cross-linking points and excess hydrogen atoms were removed. The geometric center of the formed bond was located at the center between the two cross-linking points. All sulfides were consumed for the generation of cross-linking bonds at both ends in the cross-linking process. The schematic diagram and chemical formula of the cross-linking principle were shown in Figure 4. Crosslink can be divided into self-crosslinking and crosslinking, which was distinguished by setting each molecular chain as an independent color. Here, the CNT contents of the four models were about 0 wt.%, 5 wt.%, 10 wt.% and 15 wt.%.

The geometry optimization was applied to crosslinked CNT/VNR model by conjugate gradient method with the energy and force convergence tolerance of 1 × 10^−5^ kcal/mol and 5 × 10^−4^ kcal/mol/Å [61]. Then, a 100ps 5-cycle annealing process was conducted constant volume and temperature (NVT ensemble) from 200 K to 400 K to relax the internal stress of the structure. Finally, a 250 ps dynamic relaxation under constant pressure and temperature (NPT ensemble) of 101kpa and 298 K was performed to obtain the final energy equilibrium model with reasonable vulcanization bond distances and angles.

**Figure 5:**
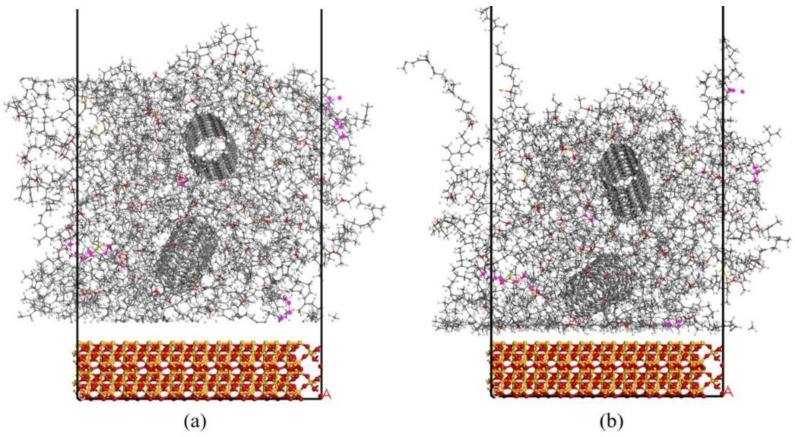
Adsorption double layer structure model of 10 wt.% CNT/VNR composite (Periodic boundary conditions are shown as black lines). (**a**) Initial state. (**b**) Equilibrium state.

Double layer and three-layer models were developed to study the interfacial properties and tribological performance. These models were constituted of CNT/VNR model and 45 × 45 × 10.8 Å^3^ SiO_2_ model. The adsorption properties between rubber matrix and SiO_2_ can be analyzed by the double layer structure as shown in Figure 5. The interfacial energy can be obtained by dynamics calculation for 150 ps under 298 K NVT ensemble with fixed bottom SiO_2_ layer. The interface interaction energy $E_{inter}$ and the interface van der Waals force energy $E_{inter-vdW}$ can be calculated by Equations (2) and (3), respectively.

(2) $$E_{inter}=E_{Total}-\left(E_{Layer1}+E_{Layer2}\right)$$

(3) $$\;\;\;\;\;\;\;\;\;\;\;\;\;\;\;\;\;\;\;\;E_{inter-vdW}=E_{Total-vdW}-\left(E_{Layer1-vdW}+E_{Layer2-vdW}\right)$$

where and are the potential energy and van der Waals energy of the entire double-layer model, respectively. and are the potential energy and van der Waals energy of the lower silicon dioxide fixed layer. and are the potential energy and van der Waals energy of the upper EUG/NR composite movable layer.

Additionally, all atoms of SiO_2_ layer needs to be unfixed before the calculation.

The frictional simulation was carried out by the confined shear calculation in Forcite module in MS based on the confined nonequilibrium molecular dynamics (NEMD) theory. The SiO_2_ slabs were initially fixed during relaxation process which including geometry optimization, annealing from 200 K to 400 K and 100 ps dynamic calculation under 298 K NVT ensemble tasks to obtain stable layer structure. After that, the bottom and top SiO_2_ slabs were unfixed and moved in the opposite direction along the *X* axis with a speed of 0.2 Å/ps for 250ps. All the friction force, layer pressure and temperature data during friction process were recorded into the trajectory files.

More details have been presented to reproduce the simulation processes. Firstly, the condensed-phase optimized molecular potentials for atomistic simulation studies (COMPASS) force field [62] was used for entire simulation work. The system is considered stable when the energy and density values of the model fluctuate less than 5% during the relaxation process. Secondly, periodic boundary conditions in x and y directions were adopted in the double layer and three-layer models to simulate properties of bulk system. Thirdly, all simulations were conducted with the time step of 1fs, the temperature and pressure controlling methods were Anderson [63] and Berendsen [64] methods, respectively. Finally, the summation methods of energy calculation were Ewald for electrostatic and Atom based for van der Waals interaction. The accuracy and buffer width of Ewald method were 1 × 10^−5^ kcal/mol and 0.5 Å. The cutoff distance, spline width and buffer width of van der Waals interaction calculations were 18.5 Å, 1 Å and 0.5 Å [61].

## 3. Results and Discussion

### 3.1. Microscopic Inherent Properties Analysis

The fractional free volume (FFV) and mean square displacement (MSD) are calculated in this part to explain and predict the enhancement of CNT on the rubber matrix.

#### 3.1.1. Fractional Free Volume

The total volume ($V_{T}$) of solid matrix can be considered as the sum of occupied volume ($V_{O}$) and free volume ($V_{F}$) based on the free volume theory [65]. The empty space represents the potential area for atoms and chains to move, therefore influencing the mechanical and thermal properties when deformation is applied to material [66]. After the relaxation process, different degrees of distortion occur in different models and it is inaccurate to discuss free volume directly. Therefore, the percentage of fractional free volume is calculated to characterize. The Connolly surface method is adopted for FFVs calculation based on Equation (4). The Connolly radius and Grid interval are 0.1 nm and 0.015 nm, respectively.

(4) $$FFV=\frac{V_{F}}{V_{T}}\times 100\%=\frac{V_{T}-V_{O}}{V_{T}}\times 100\%$$

where $V_{T}$, $V_{F}$ and $V_{O}$ represent the total, free and occupied volume of the models, respectively.

**Figure 6:**
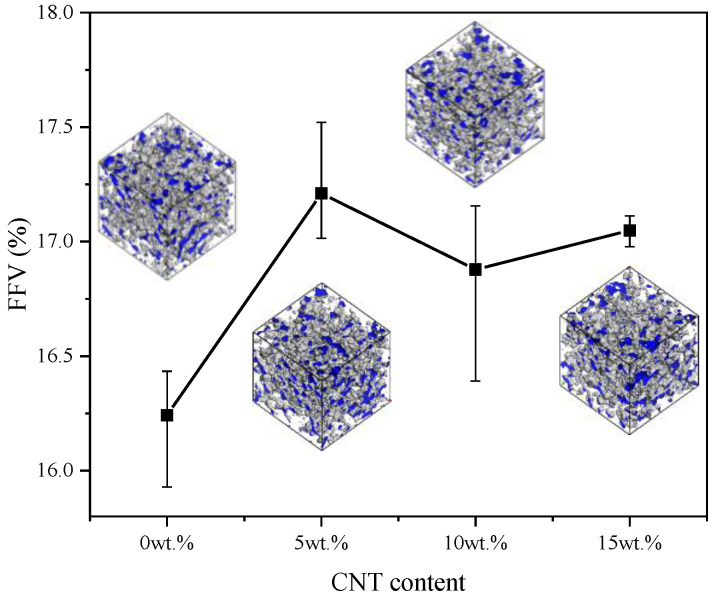
The FFVs and free volume distribution of different CNT contents CNT/VNR composites.

The results of the FFVs of pure VNR and CNT/VNR with different CNT contents are shown in Figure 6. The occupied and free volume are colored by grey and blue, respectively. It is illustrated that the addition of CNT increases the FFV of the composites. Thus, it can be inferred that CNT has an attractive effect on surrounding atoms, which leads to the agglomeration of inside atoms and formation of outside free volume. The pattern of free volume evolution with increasing CNT content can be concluded as generation-growing-convergence. During the increase of CNT content, some small free areas are generated at beginning. Then, those new formed areas become larger due to higher attractive effect from inside CNT. Finally, the grown free areas converge to form larger free volume. The molecular chains inside the concentrated rubber matrix with CNT addition show low tendency of movement, which results in less chance of internal destruction under dynamic stress. This free volume expansion at atom level is in accordance with the experimental results [26].

#### 3.1.2. Mean Square Displacement

The diffusion and movement trend of the molecular chains inside the particles can be characterized by the mean square displacement [66,67]. It indicates the statistical square of particle displacement in the system compared to the initial state, which is defined as:

(5) $$MSD=\frac{1}{3N}\overset{N-1}{\underset{i=0}{\sum }}\left({\left|R_{i}\left(t\right)-R_{i}\left(0\right)\right|}^{2}\right)$$

where $R_{i}\left(t\right)$ and $R_{i}\left(0\right)$ are the displacement vector of atom *i* at time *t* and initial time, *N* is the total number of atoms.

**Figure 7:**
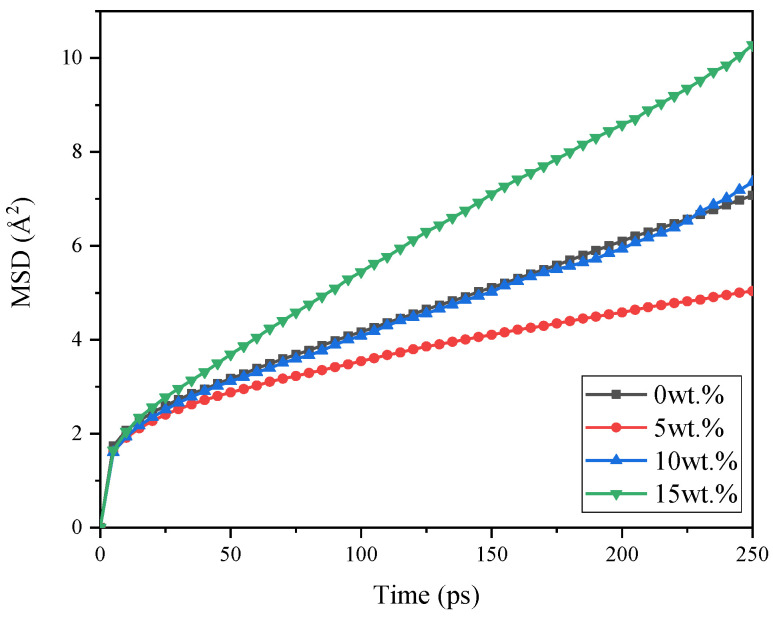
The comparatively MSD of pure VNR and CNT/VNR composites.

The MSD evolutions of pure VNR and CNT/VNR system during relaxation process are shown in Figure 7. It can be illustrated that the MSD first decrease and then increase with the addition of CNT. It can be conjectured that small amount of CNT is conductive to the aggregation of the matrix and enhancement of the composite strength due to the excellent hardness of CNT. However, agglomeration is caused by excessive nanotubes in the matrix. According to the deformation law of the hard filler-reinforced soft material, the deformation degree of the soft matrix is greater than that of overall material. Similarly, the VNR matrix surrounded by those agglomerated nanotubes shows large deformation and stress concentration, which performs higher tend of molecular movement. As a result, the CNT content should not be too high in order to avoid the agglomeration, which is consistent with related research at different scales [28].

### 3.2. Mechanical Properties Analysis

The elastic mechanical properties of the systems are analyzed by the constant strain method. Relative mechanical properties of the material including Young’s, bulk and shear modulus can be obtained by solving the stiffness matrix [68]. In MS, the stiffness matrix can be calculated by applying a series of 0.03% small tensile strains along three axes to the obtained stable models. The stable models used in this section are five independent configurations obtained in last 20 ps of relaxation process. The results are averaged and the error bars are used to express maximum and minimum among these configurations. The elements in the stiffness matrix are expressed by the following equation:

(6) $$C_{ij}=\frac{\partial^{2}U}{V\partial \varepsilon_{i}\partial \varepsilon_{j}}$$

where *U*, *V* and *ε* represent the second derivative of the deformation, unit volume and strain. For isotropic materials like rubber, the stress-strain relations are completely described by two lame constants λ and μ, which can be expressed by the elements in the stiffness matrix as [51]:

(7) $$\lambda =\frac{1}{3}\left(C_{11}+C_{22}+C_{33}\right)-\frac{2}{3}\left(C_{44}+C_{55}+C_{66}\right)$$

(8) $$\mu =\frac{1}{3}\left(C_{44}+C_{55}+C_{66}\right)$$

The Young’s modulus (*E*), bulk modulus (*K*) and shear modulus (*G*) of the systems can be further calculated based on the λ and μ results following equations below [69]:

(9) $$E=\frac{\mu \left(3\lambda +2\mu \right)}{\lambda +\mu }$$

(10) $$K=\lambda +\frac{2}{3}\mu$$

(11) G=μ

The calculation results of Young’s modulus are separated in three directions. Considering rubber as isotropic material, the modulo is calculated by Equation (12) for quantitative comparison.

(12) $$\left|E\right|=\sqrt{E_{X}^{2}+E_{Y}^{2}+E_{Z}^{2}}$$

where |*E*| is the modulo of Young’s modulus; $E_{X},E_{Y},E_{Z}$ are the Young’s modulus in *X*, *Y* and *Z* directions, respectively.

The details of bulk, shear modulus and Young’s modulo calculation results are recorded in Table 1. The results are given in the form like: minimum value~maximum value (averaged value). In addition, the increase percentage is calculated according to the averaged value. It can be concluded that the modulus increase with the increase of CNT. The bulk, shear modulus and Young’s modulo of CNT/VNR composites with 15 wt.% CNT content are 2.74, 1.09 and 5.19 GPa, which are 19.13%, 21.11% and 26.89% higher than 2.30, 0.90 and 4.09 GPa of pure VNR. This result indicates that the CNT can enhance comprehensive mechanical properties of the rubber matrix, which increases the hardness of the matrix, the resistance to shear deformation [70] and volume change [71]. The addition of CNT also allows the obtained composites to endure larger stress and suit for wider circumstance like aircraft tire production. Moreover, the increasing trend of mechanical properties of CNT/VNR composites at atom level is also conformity with experimental studies [27,28,29]. The continuous increase of CNT/VNR composites modulus can be explained by the high hardness and modulus of carbon nanotubes.

### 3.3. Interfacial Properties Analysis

The interfacial interactions of CNT/VNR composite mainly includes internal interaction of CNT- matrix and external interaction of composite-SiO_2_. Adhesion phenomenon between tire and road occurs in friction process. Hence, the double layer structure (seen in Figure 5) was developed to reveal the interface contact mechanism of the adhesion phenomenon. A clear adsorption process between composites and fixed SiO_2_ was also observed during dynamic equilibrium. The interfacial energy and atom density between CNT reinforced rubber materials and SiO_2_ are calculated in Section 3.3.1. Before dynamical friction happens, a comparatively large friction force occurs in the state of static friction due to the static adsorption. As a result, relatively large deformation occurs in this state, which brings the possibility of material damage. For nanocomposites, the dispersion condition and bonding strength of the filler inside the matrix greatly determine the damage resistance of composites during deformation [25]. In order to characterize the enhancement mechanism of CNT effects on the vulcanized natural rubber, the CNT-matrix binding energy and atom relative concentration inside nanocomposites are discussed in Section 3.3.2.

The system is considered stable when the energy fluctuation is less than 5%. All the results in this section are the average of five independent configurations in the last 20 ps after stabilization. The maximum and minimum values among the calculation results are given in the form of error bars.

#### 3.3.1. Composite-SiO_2_ Interface

**Figure 8:**
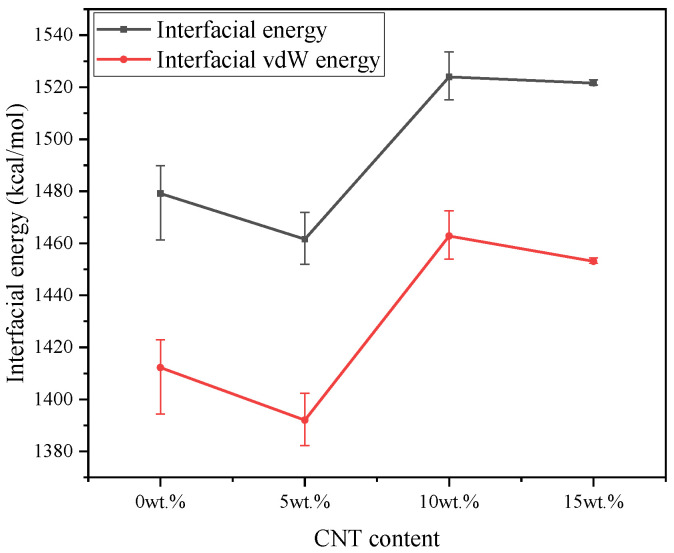
The energy of composite-SiO_2_ interface.

The interfacial energy calculation of composite-SiO_2_ interface is based on the Equations (1) and (2). The interfacial energy and van der Waals energy results of pure VNR and different CNT contents CNT/VNR composites are shown in Figure 8. It can be illustrated that the interfacial energy evolution shows nonlinear trend with the addition of CNT. The largest interfacial energy is obtained in 10 wt.% CNT/VNR composite, which means higher energy barrier needs to be break during the relative movement. This process may lead to higher temperature rise, severer atoms contact and larger static friction force. In addition, the interfacial interaction is mainly caused by the van der Waals interaction according to the contribution of van der Waals energy.

**Figure 9:**
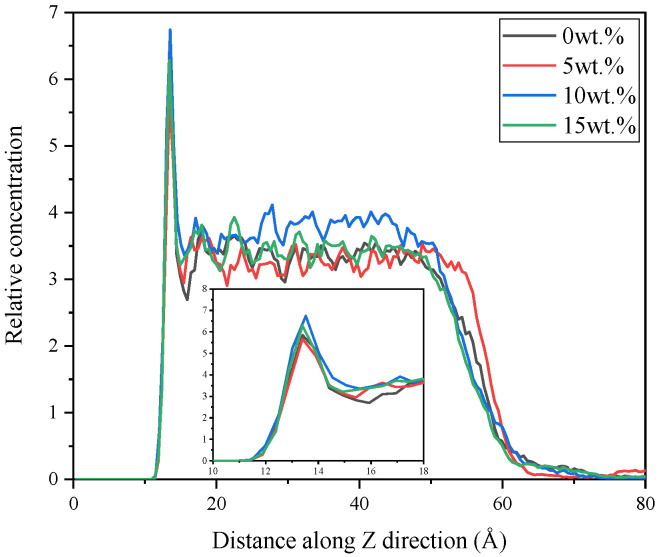
The atom relative concentration of different systems along Z direction.

The atom relative concentrations along Z direction are shown in Figure 9. It is found that the peaks of atom concentration occur in the range of 12~14 Å along Z direction. These peaks represent the aggregation degree of the atoms at the contact interface between the SiO_2_ and rubber matrix, which reflex the severity of relative motion between layers. More concentrated atoms can cause more intense friction process, which is not expected in both micro and macro applications. In the range of 50~60 Å, the relative concentration of the matrix drops rapidly, which can be explained by adsorption effect of SiO_2_ slab to the rubber matrix. It can be observed that the concentration value of 10 wt.% CNT/VNR composite in the range of 14~50 Å is higher than that of 0.5 and 15 wt.%. This phenomenon may reflect the superimposition of CNT binding and the binding weakening of excessive nanotubes. The result of interface atom concentration is consistent with the interfacial energy, which indicates that high interfacial energy can attract more internal atoms moving outward to form a high-density shell. This may be a new idea for soft materials coating preparation.

#### 3.3.2. CNT-Matrix Interface

The interfacial interactions between CNT and rubber matrix are discussed by using the models as same as Section 3.2. The binding energy calculations follow similar pattern of interfacial energy calculations, which is expressed by Equations (13) and (14).

(13) $$E_{b}=-\left(E_{T}-E_{r}-E_{CNT}\right)$$

(14) $$E_{b-vdW}=-\left(E_{T-vdW}-E_{r-vdW}-E_{CNT-vdW}\right)$$

where $E_{b}$, $E_{T}$, $E_{r}$, $E_{CNT}$ and $E_{b-vdW}$, $E_{T-vdW}$, $E_{r-vdW}$, $E_{CNT-vdW}\;$ represent the potential and van der Waals energy of binding, total system, rubber and CNT, respectively.

**Figure 10:**
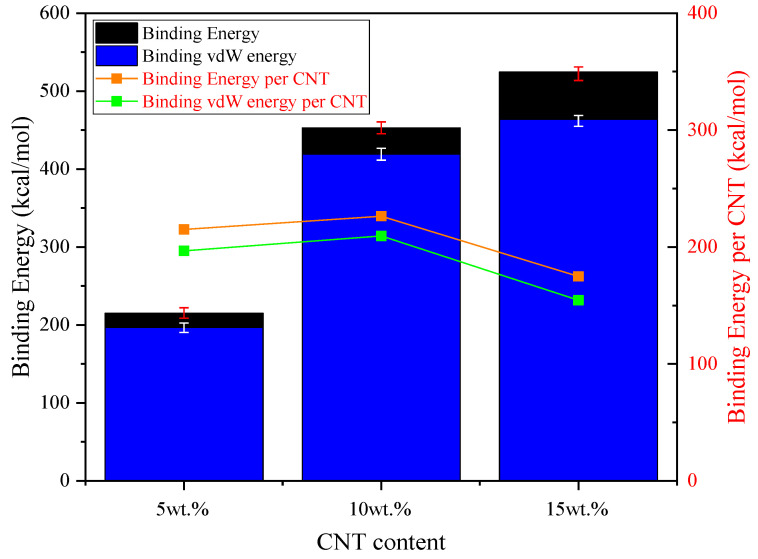
The binding energy of CNT/VNR composites with different CNT contents.

The results of total binding energy and binding energy per CNT are shown in Figure 10. It can be indicated that the binding energy between rubber matrix and CNT increases with the increase of CNT. It is easily to understand that more nanotubes have more contact area with rubber matrix and the superimposition effect of nanotubes binding mentioned before causes greater total binding energy of higher CNT content. However, the binding energy per CNT no longer shows linear growth but decrease when excessive nanotubes exist in the rubber matrix. This binding weakening phenomenon may be caused by the agglomeration of nanotubes. These agglomerated nanotubes begin to compete for atoms located around their geometric center when the superposition effect of CNT exceeded its maximum, which decreases the binding energy per CNT. Macroscopically, it is reflected by the decrease of mechanical properties like tensile strength, elongation at break and so on [7,28,29]. In addition, it can be observed that the van der Waals binding energy constitutes most part of the total binding energy, which proves the van der Waals interaction is the main factor of binding between CNT and rubber matrix.

**Figure 11:**
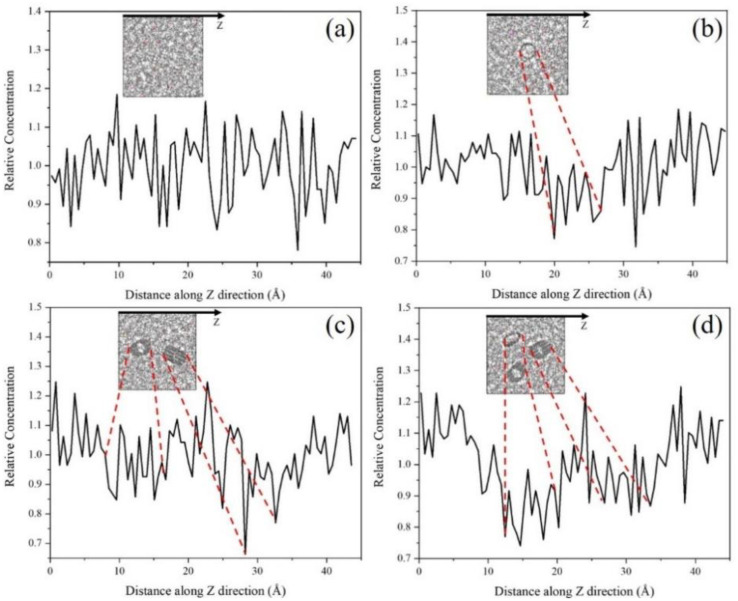
Axial density distribution of CNT/VNR composites with different CNT contents. (**a**) Pure VNR. (**b**) 5 wt.%CNT/VNR. (**c**) 10 wt.%CNT/VNR. (**d**) 15 wt.%CNT/VNR.

The density distributions of rubber matrix inside the composite are also obtained as shown in Figure 11. Obvious peaks can be observed at the CNT-rubber matrix interface, which indicates the attractive effect of CNT on surrounding atoms. This is consistent with the conclusions obtained in Section 3.1 and Section 3.2, which explains the strengthening mechanism of CNT on the rubber matrix. In addition, higher atomic densities are observed between two different nanotubes than that on both sides, which also indicates that the attraction of CNT has a superimposed effect. The relatively low atom density in the 15 wt.%CNT/VNR reflects the compete relationship between excessive nanotubes. This attraction-superposition-competition dynamic changing process caused by the increase of CNT content may be one of the main reasons for the changes of mechanical and tribological properties in actual applications.

In conclusion, both the internal binding and external interaction are influenced by CNT. The dynamic process of attraction-superposition-competition is inferred by molecular simulations. The friction performance is preliminarily predicted based on the interfacial calculation results. In order to verify the rationality of predictions, the tribological properties are discussed below.

### 3.4. Tribological Properties and Mechanism

The COF is computed in this section for evaluating the tribological properties of the different CNT contents CNT/VNR composites. The details of calculated COF are given in the form like: minimum value~maximum value (averaged value) and listed in the Table 2.

Results indicate that the COF keep rising with the addition of CNT. However, the growth rate drops significantly when CNT content changes from 10 wt.% to 15 wt.%. The discrepancy of COF caused by the surface roughness can be ignored at atomic level in our simulations. The fiction performance of the composite can no longer be simply judged by the interfacial energy but a combined result of mechanical and interfacial properties. On one hand, the increasing mechanical modulus prevent large shear deformation of the composite, which makes the composite become more stable during the friction process. The atoms around the friction surface tend to show lower activity in this stable composite, which achieves better equilibrium adsorption between the composite and the SiO_2_ slab. That’s the reason why the COF increase with the addition of CNT. On the other hand, the friction performance also effected by the interfacial energy. Although the CNT/VNR composites with lower CNT content are harder to achieve well equilibrium adsorption state during friction process, they still have larger interfacial energy (especially the 10 wt.% CNT/VNR) to offset the inadequate surface interaction caused by insufficient mechanical properties. Thus, the COF of composites are not show a linear trend of increasing like the modulus, but increasing trend of slowing down.

**Figure 12:**
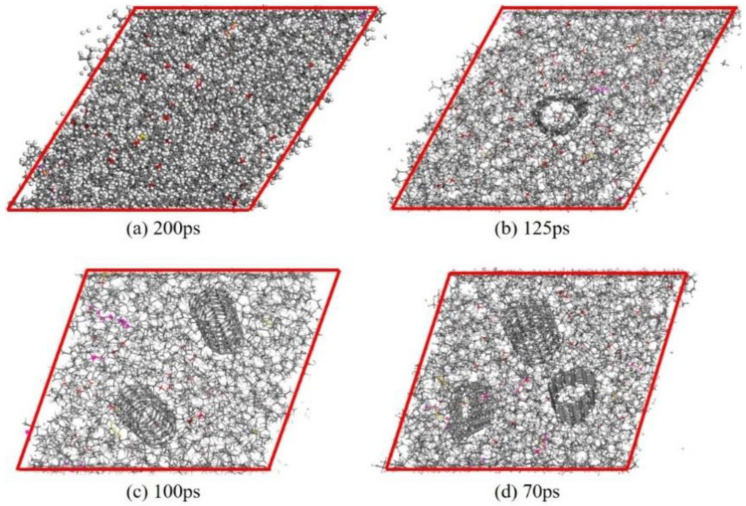
Deformation state of different CNT/VNR composites and time at the beginning of slipping. (**a**) Pure VNR. (**b**) 5 wt.%CNT/VNR composite. (**c**) 10 wt.%CNT/VNR composite. (**d**) 15 wt.%CNT/VNR composite.

**Figure 13:**
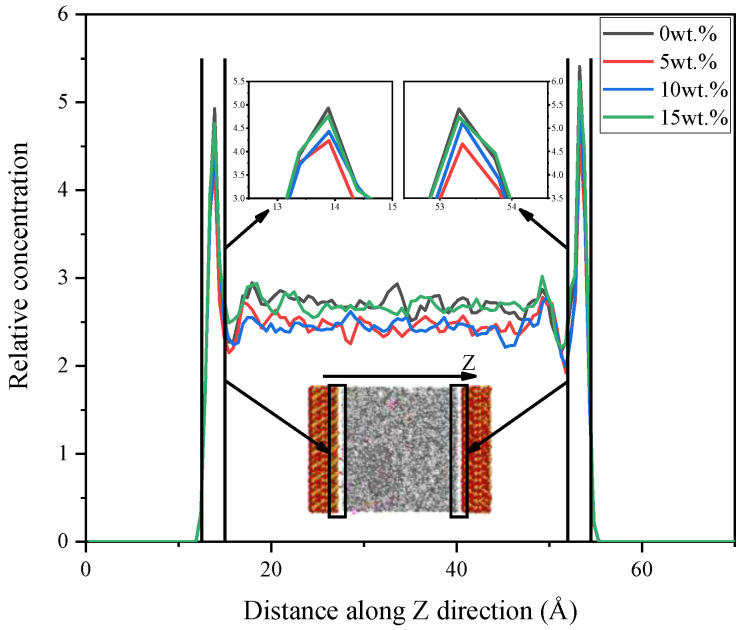
Relative atom concentration of different CNT/VNR composites.

The slip phenomenon is observed during friction process and relative atom concentration along Z direction are further discussed for the tribological properties. During the friction process, the shear deformation of the composite is observed in the beginning. Then, the deformation of the composite reaches its maximum state and the slip phenomenon can be observed afterward. The slip mechanism may be caused by the relative movement, which breaks the energy barrier formed by interfacial interaction. Therefore, the state of the composite and the time of slip phenomenon occurs can be used to reveal the mechanism of the COF evolution. The relative concentration reflects the severity of the friction. More concentrated atoms cause more intense friction process. The state of different CNT/VNR composites and the time when slip phenomenon occurs are shown in Figure 12. The relative atom concentration along Z direction is shown in Figure 13. It can be observed that CNT/VNR composites with higher CNT content have lower shear deformation and the slip phenomenon occurred earlier. This enhanced property prevents the damage of composite during friction and allows the composite to reach stable dynamic friction sooner. The relative concentration indicates that the atoms appear to gather on the rubber matrix rather than friction interface due to the enhanced binding properties of CNT, which can reduce the intensity of friction. The maximum decrease of the atom concentration is observed in the 5 wt.% CNT/VNR composite, which are 14.3% and 13.8% lower than that of pure VNR on different friction interfaces. However, this enhancement can be attenuated by excessive nanotubes, which may be caused by the weakened binding properties. As a result, this impact needs to be noticed in actual prescription design of CNT reinforced rubber materials.

## 4. Conclusions

MD simulations were performed to reveal the influence of CNT content on the CNT/VNR composites and the enhanced mechanism. The results are consistent with the present research, which can be used to reveal the enhanced mechanism of CNT. The following conclusions are highlighted from the results.

(1) Molecular models for the CNT/VNR composites with different CNT content were developed with considering the distribution of different sulfur bonds. The FFV and MSD were discussed preliminarily. The FFV evolution with CNT addition is summarized as three stages, which are generation, growing and convergence.
(2) The bulk, shear and Young’s modulus were calculated by constant strain method to evaluate the mechanical properties of the composites. Approximately linear increase trends were observed in all modulus. The largest bulk, shear and Young’s modulus occurred in the 15 wt.% CNT/VNR composite, which were 19.13%, 21.11% and 26.89% higher than that of pure VNR, respectively. The mechanism of these improved mechanical properties can be explained by the high strength of CNT.
(3) The binding energy of CNT-matrix interface and the interfacial energy of composite-SiO_2_ interface were obtained, respectively. The largest interfacial energy was obtained in 10 wt.% CNT/VNR composite. Thus, a dynamic attraction-superposition-competition process is concluded to reveal the reinforced mechanism of CNT on the rubber matrix. Both the binding and interfacial interactions are mainly produced by the van der Waals interaction.
(4) The COF and relative concentration at the friction interface were calculated to discuss the tribological properties of CNT reinforced VNR composite. The COF shows a nonlinear trend of increasing. Based on the mechanical and interfacial results, the friction performance is inferred to be a combined consequence of mechanical and interfacial properties. Although the enhanced COF is expected in the production of aircraft tires, the addition of CNT can cause more intense friction process according to the relative concentration results. Therefore, this impact needs to be noticed in the actual design of prescription.

## Figures and Tables

**Table 1:** Bulk, Shear modulus and Young’s modulo results and increase percentage compared to pure VNR.

CNT Content (wt.%)	Bulk Modulus (GPa)	Increase (%)	Shear Modulus (GPa)	Increase (%)	Young’s Modulo (GPa)	Increase (%)
0	2.22~2.34 (2.30)	0	0.88~0.94 (0.90)	0	4.01~4.15 (4.09)	0
5	2.35~2.51 (2.42)	5.22	0.94~1.00 (0.96)	6.67	4.23~4.59 (4.42)	8.07
10	2.50~2.77 (2.61)	13.48	1.01~1.06 (1.03)	14.44	4.70~4.94 (4.83)	18.09
15	2.65~2.85 (2.74)	19.13	1.05~1.12 (1.09)	21.11	4.99~5.31 (5.19)	26.89

**Table 2:** COF details of different CNT contents CNT/VNR composites.

CNT Content (wt.%)	COF	Increased Percentage (%)
0	1.017~1.049 (1.033)	0
5	1.037~1.118 (1.078)	4.36
10	1.097~1.192 (1.144)	10.75
15	1.104~1.207 (1.155)	11.81

## Data Availability

The data presented in this study are available on request from the corresponding author.
